# The molecular mechanism of photochemical internalization of cell penetrating peptide-cargo-photosensitizer conjugates

**DOI:** 10.1038/srep18577

**Published:** 2015-12-21

**Authors:** Takashi Ohtsuki, Shunya Miki, Shouhei Kobayashi, Tokuko Haraguchi, Eiji Nakata, Kazutaka Hirakawa, Kensuke Sumita, Kazunori Watanabe, Shigetoshi Okazaki

**Affiliations:** 1 Department of Medical Bioengineering, Okayama University, 3-1-1 Tsushimanaka, Okayama 700-8530, Japan; 2 Advanced ICT Research Institute Kobe, NICT, 588-2 Iwaoka, Iwaoka-cho, Nishi-ku, Kobe 651-2492, Japan; 3 Institute of Advanced Energy, Kyoto University, Gokasho,Uji, Kyoto 611-0011, Japan; 4 Department of Applied Chemistry and Biochemical Engineering, Graduate School of Engineering, Shizuoka University, Johoku 3-5-1, Naka-ku, Hamamatsu 432-8561, Japan; 5 Department of Medical Spectroscopy, Hamamatsu University School of Medicine, 1-20-1 Handayayama, Higashi-ku, Hamamatsu, 431-3192, Japan

## Abstract

In many drug delivery strategies, an inefficient transfer of macromolecules such as proteins and nucleic acids to the cytosol often occurs because of their endosomal entrapment. One of the methods to overcome this problem is photochemical internalization, which is achieved using a photosensitizer and light to facilitate the endosomal escape of the macromolecule. In this study, we examined the molecular mechanism of photochemical internalization of cell penetrating peptide-cargo (macromolecule)-photosensitizer conjugates. We measured the photophysical properties of eight dyes (photosensitizer candidates) and determined the respective endosomal escape efficiencies using these dyes. Correlation plots between these factors indicated that the photogenerated ^1^O_2_ molecules from photosensitizers were highly related to the endosomal escape efficiencies. The contribution of ^1^O_2_ was confirmed using ^1^O_2_ quenchers. In addition, time-lapse fluorescence imaging showed that the photoinduced endosomal escape occurred at a few seconds to a few minutes after irradiation (much longer than ^1^O_2_ lifetime), and that the pH increased in the endosome prior to the endosomal escape of the macromolecule.

The therapeutic efficacy of many drug delivery strategies is often limited by the inefficient transfer of cargo macromolecules such as proteins and nucleic acids to the cytosol consequential to their endosomal entrapment^1^,^2^. One of the methods to overcome this problem is to use a photosensitizer and light to facilitate the endosomal escape of the macromolecules^3^,^4^,^5^,^6^,^7^,^8^, termed photochemical internalization (PCI). It has been considered that in this technique, the endosomes are disrupted by reactive oxygen species generated photo-dependently from photosensitizers^9^,^10^,^11^,^12^. However, the correlations between endosomal escape efficiency and the photosensitizing reactions of various photosensitizers have not been systematically examined. Thus, the properties of photosensitizers important for PCI need to be clarified through the use of multiple photosensitizers.

To analyze the mechanism of PCI, a photosensitizing RNA carrier molecule was used in this study. This carrier molecule can bind to an RNA and deliver it into a cell by the PCI strategy, whereby the carrier/RNA complexes are first entrapped within endosomes, and then photo-dependently escape the endosomes. The photosensitizing RNA carrier molecule consists of a photosensitizer and TatU1A, a fusion protein of HIV TAT-derived cell-penetrating peptide (CPP) and human U1A-derived RNA-binding protein^13^,^14^,^15^. An advantage to the use of a photosensitizer attached to the TatU1A protein for studying the PCI mechanism is that the localization of photosensitizers (~1 kDa) is strongly affected by the larger protein moiety (~16 kDa). Thus, the difference of intrinsic “cellular localization” among various photosensitizers can virtually be ignored, and focus can be placed on the “photosensitizing reaction” of the photosensitizers.

In this study, we measured the photophysical properties of eight dyes as photosensitizer candidates, and investigated which property is most related to effective photoinduced endosomal disruption using TatU1A-dye (TatU1A-photosensitizer) conjugates. The fluorescence quantum yield (φ) and ^1^O_2_ quantum yield (φ_Δ_) were measured as the photophysical dye properties. In addition, the photo-dependent heat generation efficiency was approximately estimated by the parameter (1 − φ − φ_Δ_). The influence of ^1^O_2_ quenchers was utilized to investigate the PCI mechanism. Furthermore, time-lapse images of the photoinduced endosomal disruption events were analyzed.

## Results

### Photo-dependent cytosolic RNA internalization using various TatU1A-dyes

**Figure 1:**
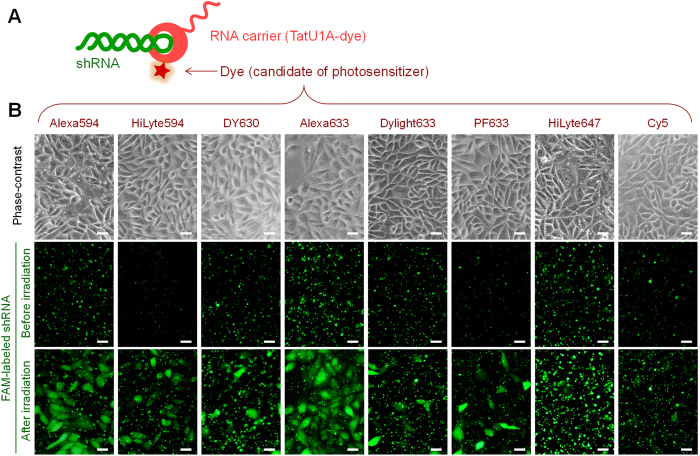
Photoinduced RNA internalization by CHO cells. (**A**) Complex of the RNA carrier-TatU1A-dye with FAM-labeled short hairpin RNA (shRNA). (**B**) Phase contrast and FAM fluorescence images of the cells after incubation with TatU1A-dye/FAM-labeled RNA complex and followed by photostimulation. Scale bars indicate 20 μm.

Various TatU1A-dye conjugates (Fig. 1A) were used to attempt cellular RNA delivery and photoinduced cytoplasmic dispersion (or endosomal escape) of the RNA. Eight dyes, that absorb yellow to red light (550–650 nm), were used as candidates for photosensitizers for the PCI strategy. Since these dyes can be excited by similar excitation wavelengths, endosomal escape efficiencies using these dyes were compared under the same photostimulation conditions (wavelength, light intensity, and irradiation time) (Table 1). Photo-dependent cytoplasmic dispersion of the RNA was efficiently induced with TatU1A-Alexa633 and TatU1A-Alexa594 (Fig. 1B), indicating that these dyes can work as photosensitizers when they are attached to the TatU1A protein. In contrast, several dyes, such as DyLight 633 and Cy5, induced only minimal photo-dependent endosomal RNA escape.

### Contributions of the photophysical properties of the dyes to endosomal escape

**Figure 2:**
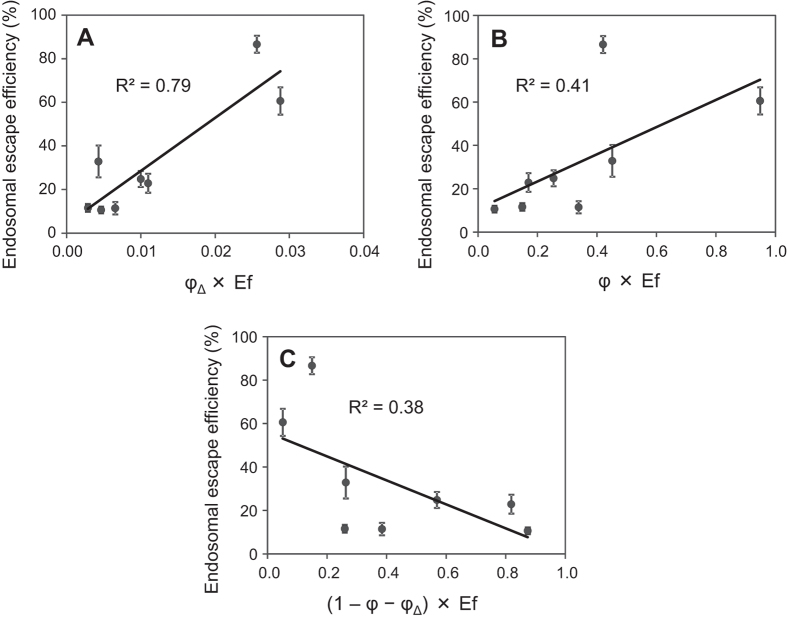
Relationship between endosomal escape and photophysical parameters of phorosensitizers. Correlations between photoinduced endosomal escape efficiencies of the TatU1A-dye/RNA complexes and [φ_Δ_ × Ef] (**A**), [φ × Ef] (**B**), or [(1 − φ − φ_Δ_) × Ef] (**C**) of each dye. The φ_Δ_ and φ values were measured in octanol. Data represent means ± SD, n = 5.

To identify the key characteristics of the dyes that contribute to photo-dependent endosomal escape of the cargo RNA, we investigated the phenomena after excitation of the dyes by light. The major phenomena after dye excitation are fluorescence emission, heat generation, and ^1^O_2_ generation. First, the ^1^O_2_ quantum yields (φ_Δ_) of these dyes were estimated by measuring the near-infrared phosphorescence of ^1^O_2_ at around 1270 nm. Photoinduced ^1^O_2_ generation from the dyes was detected for some dyes upon being dissolved in 1-octanol (Table 1), though this was hardly detected in water, probably because of the weak phosphorescence of ^1^O_2_ in water. Octanol, having a long hydrophobic carbon chain with a small hydrophilic hydroxyl group, seems to provide an environment similar to that in membrane lipids. The relative extent of ^1^O_2_ photogenerated from each photosensitizer in the endosomal escape experiment was estimated by the formula [φ_Δ_ × Ef], in which Ef is the efficacy of excitation of each photosensitizer. The [φ_Δ_ × Ef] value was highly correlated with the endosomal escape efficiencies of TatU1A-dye/RNA complexes (R^2^ = 0.79) (Fig. 2a), indicating that photoinduced ^1^O_2_ generation from dyes is required for endosomal escape of the dye-conjugate molecules.

In contrast, the relative extent of the fluorescence quantum yield of each dye in octanol did not significantly correlate with the endosomal escape efficiency of the TatU1A-dye/RNA complex (R^2^ < 0.5) (Fig. 2B). The fluorescence quantum yield of each dye in water also did not correlate with the endosomal escape efficiency (Supplementary Fig. S1A).

After excitation of these dyes, the absorbed light energy would be used mainly for fluorescence, ^1^O_2_ generation, and heat generation. Since phosphorescence was not detected from these dyes, the value [1 – (fluorescence quantum yield φ) – (singlet oxygen quantum yield φ_Δ_)] is likely to represent a kind of quantum yield for heat, that is, [heat energy generated from a dye]/[total light energy absorbed by the dye]. To consider the contribution of heat generation from dyes, the correlation between [(1 − φ − φ_Δ_) × Ef] and the endosomal escape efficiency was evaluated. Figure 2C shows that there was no significant correlation between these measures, suggesting that heat generated from dyes does not contribute significantly to the endosomal escape of the carrier/RNA complex. These results (Fig. 2) indicate that photo-dependent endosomal escape is most highly related to ^1^O_2_ among the three major phenomena (fluorescence, heat, and ^1^O_2_) occurring after dye excitation.

We note that the majority of reported PCI experiments have utilized photosensitizers that were not covalently attached to carrier-cargo complexes^5^,^6^,^7^,^16^,^17^,^18^. In contrast, in this study, we examined a photosensitizer that was covalently linked to the carrier protein. If this study had been performed using unlinked photosensitizers, we could not have concluded that only the photoinduced ^1^O_2_ generation of the photosensitizers was important to effect endosomal escape of the cargo, because endosomal escape efficiency must also be affected by the specific cellular localization of each photosensitizer. However, here the influence of the localization was minimized through the use of photosensitizers attached to a protein that always delivers each photosensitizer into endosomes.

### PCI using TatU1A-rose bengal and a shRNA cargo molecule

**Figure 3:**
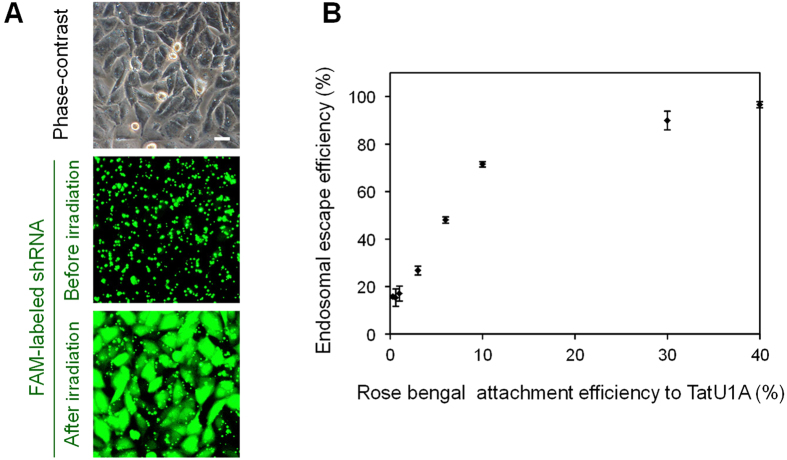
PCI using the TatU1A-rose bengal/RNA complex. (**A**) Photoinduced endosomal escape of the FAM-labeled shRNA using TatU1A-rose bengal (rose bengal attachment efficiency was 30%). Scale bars indicate 20 μm. (**B**) Correlation between the photoinduced endosomal escape efficiency of the TatU1A-rose bengal/RNA complex and the rate of attachment of rose bengal to TatU1A. To induce endosomal escape, the cells were irradiated at 530–550 nm, 930 mW/cm^2^ for 11 s. Data represent means ± SD, n = 3.

To further investigate the contribution of photoinduced ^1^O_2_ generation to endosomal escape, we performed PCI using TatU1A-rose bengal and the FAM-labeled shRNA as the cargo molecule. Rose bengal is a well-known photosensitizer with a very high ^1^O_2_ quantum yield (φ_Δ_ = 0.86 in EtOH^19^). We prepared several TatU1A-rose bengal conjugates with different rose bengal attachment efficiencies to the TatU1A protein (0–40%), and investigated the correlation between the attachment efficiency and endosomal escape efficiency. Photoinduced endosomal escape of the FAM-labeled shRNA using TatU1A-rose bengal was confirmed as shown in Fig. 3A. Figure 3B shows that the endosomal escape efficiency linearly correlated with the rose bengal attachment efficiency until the latter reached 10%. At greater attachment efficiencies, the endosomal escape efficiency approached a plateau. Since the light energy absorbed by rose bengal is primarily utilized for ^1^O_2_ generation, this result suggests that endosomal escape correlates to photoinduced ^1^O_2_ generation.

### Influences of the ^1^O_2_ quenchers

**Figure 4:**
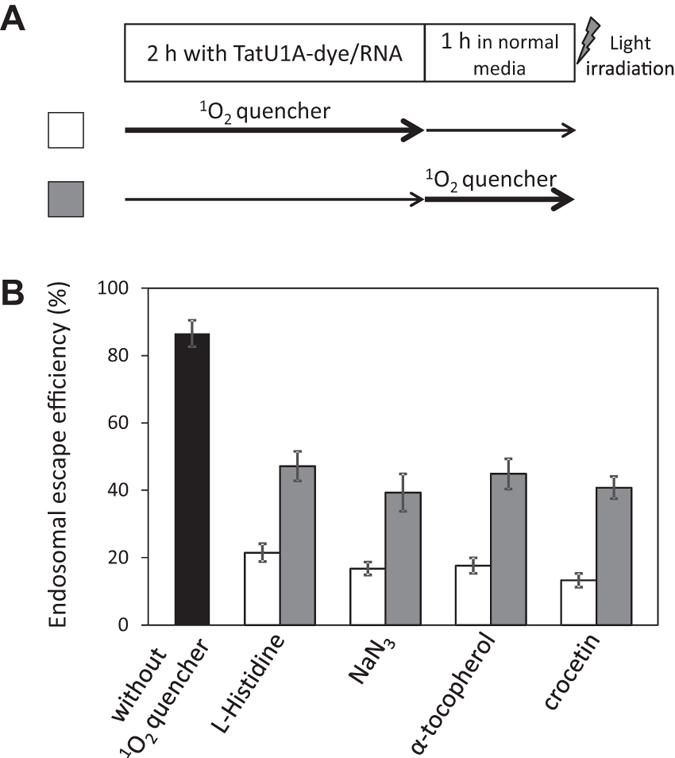
Influences of ^1^O_2_ quenchers on endosomal escape efficiency. Each ^1^O_2_ quencher was added to the cells simultaneous with (white bars) or subsequent to (gray bars) the treatment with the complex. The timing of ^1^O_2_ quencher addition to the cells is shown in the lower panel. Data represent means ± SD, n = 3.

To further confirm the contribution of ^1^O_2_, the influences of ^1^O_2_ quenchers on the endosomal escape efficiency were examined. We used two hydrophilic quenchers (histidine and NaN_3_) and two lipophilic quenchers (α-tocopherol and crocetin). These ^1^O_2_ quenchers were added (i) with the same timing as the 2 h incubation with the TatU1A-Alexa633/RNA complex, or (ii) during the 1 h following complex incubation (Fig. 4, lower panel). All of the quenchers added at both times (i) and (ii) downregulated the endosomal escape efficiency of the TatU1A-Alexa633/RNA complex (Fig. 4). These results demonstrated significant correlation between photoinduced ^1^O_2_ generation and endosomal escape efficiency of the CPP-cargo-photosensitizer conjugate.

### Imaging of photoinduced endosomal escape

**Figure 5:**
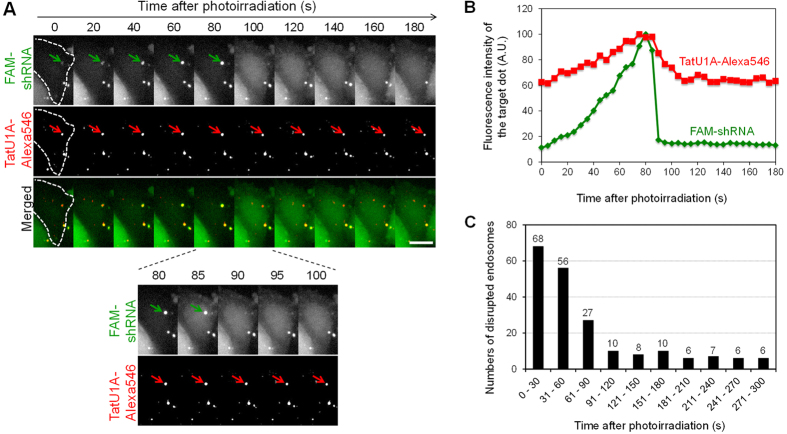
Time-lapse images and analyses of photoinduced endosomal disruption. (**A**) Time-lapse images of endosomal disruption after photoirradiation. The arrow shows the endosome that will be disrupted. Scale bars indicate 10 μm. (**B**) FAM and Alexa546 fluorescence intensities in the endosomes indicated by the arrows in (**A**). Background FAM or Alexa546 fluorescence intensity at the first time point (0 s) was subtracted from all data. Fluorescence intensities were normalized to the maximum fluorescence intensities of each of FAM and Alexa546. (**C**) Numbers of disrupted endosomes at each time after photoirradiation. In total, 204 endosomes in 159 cells were examined.

To examine the mechanism of photoinduced endosomal cargo escape, time-lapse imaging was performed. In this experiment, CHO cells were treated with the complex of TatU1A-Alexa546 and FAM-labeled shRNA and then were photoirradiated. Time-lapse images of FAM-shRNA and TatU1A-Alexa546 after photoirradiation revealed that FAM fluorescence at the sites of endosomal dots increased with time (Fig. 5A, green arrows, and Fig. 5B) and then suddenly decreased to the original level within a few seconds (Fig. 5A,B, at 85–90 s). This rapid decrease in FAM fluorescence at the endosomal dot was always accompanied by the dispersion of FAM fluorescence into the cytosol, indicating that most of the FAM-labeled cargos were escaped from the disrupted endosomes. After the endosomal escape, the FAM fluorescence intensity in the disrupted endosome was almost the same as that in the surrounding area. Among the 204 disrupted endosomes, 151 (74%) were disrupted at 0–90 s after photoirradiation (Fig. 5C). Notably, the increase of FAM fluorescence prior to endosome disruption was commonly observed for the endosomes which could be analyzed using a single particle tracking method (n = 26). We speculated that the increase of FAM fluorescence is most likely to indicate a pH increase in the endosome. The finding that the fluorescence of the FAM-labeled shRNA was much stronger at pH7.2 (which is similar to the cytosolic pH) than at pH5.5 (similar to the endosomal pH) (Supplementary Fig. S2) supports this notion.

In contrast to the FAM fluorescence, significant levels of Alexa546 fluorescence were still detected at the sites of the disrupted endosomes (Fig. 5A, red arrows, and Fig. 5B), indicating that most of the carrier molecules dissociated from the dispersed cargoes and remained at the original site of localization. This phenomenon might be partially explained by the fact that the Tat peptide has a strong affinity to endosomal membranes. It was recently reported that endosome disruption around exogenous materials triggers autophagy within about 5 min^20^,^21^, with ubiquitinated proteins on the remnants of the disrupted endosomes being the plausible autophagy targets^22^. Thus, rapid dissociation of the cargos from their carriers immediately after endosomal disruption might contribute the overall efficiency of cargo internalization into the cytosol in our present method.

## Discussion

In this study, we investigated the molecular mechanism of photo-dependent endosomal disruption using CPP-cargo-photosensitizer conjugates. We employed eight dyes, such as Alexa Fluor 633 and DY630, as photosensitizers. These are not the photosensitizers used in most previous PCI studies, which utilized porphyrin-related molecules such as meso-tetraphenyl porphyrin disulphonate (TPPS_2a_)^23^,^24^,^25^, disulfonated aluminum phthalocyanine (AlPcS_2a_)^26^, and disulfonated tetraphenyl chlorin (TPCS_2a_), the latter of which has been reported as a clinically suitable PCI photosensitizer^27^. However, in this study, the photosensitizers were required to have a maleimide group for the reaction with the thiol group of the TatU1A protein, and at present, porphyrin photosensitizers with maleimide groups are not commercially available. Thus, eight commercially available dyes, which have similar excitation wavelengths to each other, were employed as photosensitizers in this study. Compared to porphyrin, the dyes used here are weak photosensitizers (*i.e*., their ^1^O_2_ quantum yields are much lower than that of porphyrin). However, we demonstrated that certain of these weak photosensitizers could work effectively in PCI when attached to the Tat fusion protein.

**Figure 6:**
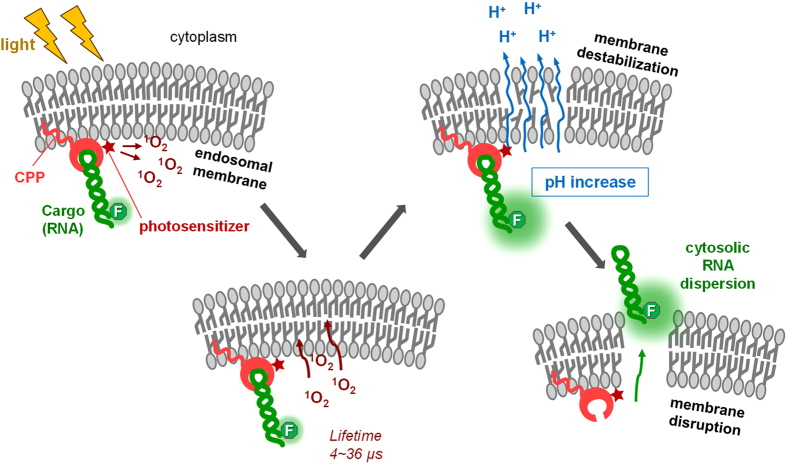
Schematic illustration of the endosomal disruption process using the cell penetrating peptide (CPP)-cargo-photosensitizer conjugate suggested by this study.

Correlation plots between endosome escape efficiencies and the photophysical properties of each photosensitizer candidate indicated that the generated ^1^O_2_ from the photosensitizer was highly correlated to the endosomal escape efficiency. The ^1^O_2_ lifetime in lipid bilayers (12-36 μs)^28^ differs from that in water (4.2 μs)^29^. Therefore, the localization of photosensitizers affects the efficacy of the ^1^O_2_ produced. Photosensitizers might be located close to the membrane as presented in Fig. 6, or might also be partly embedded into the endosomal membranes to an extent depending on the chemical properties of the photosensitizers. However, in this study, the photosensitizers were not free, and the localizations of the photosensitizers must be highly affected by the linked TatU1A that is much larger than the photosensitizers themselves. Thus, the localizations of the individual photosensitizers are presumed to be similar. Furthermore, the difference between the lifetime of ^1^O_2_ generated near the membrane and that within the membrane seems to be diminished because ^1^O_2_ is likely to quickly enter and exit the membrane. The time to traverse a root mean-square distance of 40 Å, the thickness of lipid bilayer, is ~2 ns^30^. This might be the reason for the finding that the endosomal escape efficiencies of the carrier/RNA complexes were highly correlated with the [φ_Δ_ × Ef] values without considering the localization of each photosensitizer.

The contribution of ^1^O_2_ was also indicated using ^1^O_2_ quenchers. We used four different quenchers, two hydrophilic and two hydrophobic. It was notable that the effects of all the quenchers were very similar despite their different properties. The result that both hydrophilic and hydrophobic quenchers decreased endosomal escape efficiency suggested that the photosensitizers (dyes) of the TatU1A-dye molecules were partly embedded into the endosomal membrane and affected by the hydrophobic quenchers, and partly within the endosomal matrix and thus affected by hydrophilic quenchers as well.

Time-lapse imaging of the endosomal escape indicated that photostimulation induced a pH increase in the endosome followed by endosomal disruption. Imaging of photoinduced endosomal release of macromolecules was also attempted by de Bruin *et al.*^31^. They demonstrated PCI of macromolecules such as dextran labeled with Alexa Fluor 488 and a polyethyleneimine (Alexa Fluor 488)/DNA (Cy5) complex, using meso-tetraphenyl porphyrin disulphonate (TPPS_2a_) as a photosensitizer, which was not covalently linked to the macromolecules. From this, they clearly showed the release dynamics of the macromolecules, but did not mention any pH increase prior to the endosomal escape. Thus, we believe that the pH increase prior to endosomal escape observed in this study represents a novel finding.

Overall, the results demonstrated in this study suggest that the photoinduced endosomal disruption occurred as follows (Fig. 6): (i) the endosomal membrane was destabilized; (ii) protons were released from the endosome, elevating internal endosome pH; and (iii) the endosomal membrane disrupted and the cargo RNA dispersed from the endosome into the cytosol. The timescale for the photoinduced endosomal disruption (a few seconds to a few minutes) was much longer than the ^1^O_2_ lifetime (4.2 μs in water and 19 μs in octanol^29^). Thus, a process(es) must exist after ^1^O_2_ generation until endosomal disruption that requires a few seconds to a few minutes to complete. This might be a destabilization process induced by membrane lipids damaged by ^1^O_2_. The hidden process between ^1^O_2_ generation and endosomal disruption needs to be clarified by future experiments.

## Methods

### Preparation of TatU1A-dye conjugates

The RNA carrier protein TatU1A, which has a C-terminal Cys residue, was prepared as described previously^15^. The purified TatU1A protein and a dye with a thiol-reactive maleimide group were mixed in a buffer containing 50 mM HEPES-KOH (pH 7.5), 100 mM (NH_4_)_2_SO_4_, 150 mM imidazole, and 20% glycerol, and then incubated at 25 °C for 1 h. The dyes used here were Alexa Fluor 594 (Life Technologies, Carlsbad, CA), HiLyte Fluor 594 (AnaSpec, Fremont, CA), Alexa Fluor 633 (Life Technologies), DyLight 633 (Pierce, Rockford, IL), DY 630 (Dyomics, Jena, Germany), Promo Fluor 633 (PromoKine, Heidelberg, Germany), HiLyte Fluor 647 (AnaSpec), Cy5 (GE Healthcare Life Sciences, Tokyo, Japan), and rose bengal, which was synthesized as described in supporting information. The dye-attached TatU1A molecules (TatU1A-dye) were purified in a Centri-Sep spin column (Princeton Separations, Freehold Township, NJ) equilibrated with T buffer [20 mM HEPES-KOH (pH 7.4), 115 mM NaCl, 5.4 mM KCl, 1.8 mM CaCl_2_, 0.8 mM MgCl_2_, and 13.8 mM glucose]. Protein concentration was determined using a Protein Assay Kit (Bio-Rad, Berkeley, CA). Labeling efficiencies of the carrier proteins were calculated by measuring the absorbance of the respective dyes. In all experiments, labeling efficiencies were adjusted to 40% using separately prepared unlabeled carrier proteins.

### FAM-labeled shRNA

A FAM-labeled shRNA was purchased from JBioS (Saitama, Japan). The shRNA sequence used was as follows: 5′- GAU UAU GUC CGG UUA UGU ACA UUG CAC UCC GUA CAU AAC CGG ACA UAA UCdT dT -3′ (the U1A binding sequence^32^ is underlined). This represents a non-specific shRNA in normal mammalian cells, but contains an anti-luciferase sequence. A fluorescent dye (FAM) was attached at the 3′-end of the shRNA. The shRNA was annealed by incubation at 85 °C for 1 min to remove secondary structure followed by slow annealing (−1 °C/s) to 4 °C.

### Measurement of the ^1^O_2_ quantum yields of dyes

The ^1^O_2_ generation quantum yields of each dye were calculated by comparing the emission intensities of the singlet oxygen photosensitized by each dye at 1270 nm using methylene blue as the standard. In detail, a nanosecond pulsed Nd:YAG laser pumped dye (DCM) laser (500 Hz, pulse width 7 ns, 50 mW) was used for the excitation source, and the emission from a singlet oxygen from the dye was collected by the bundle fiber and detected by the near infrared-photomultiplier tube (NIR-PMT) (H10330B-45, Hamamatsu Photonics K.K., Hamamatsu, Japan) through the band-pass filter (1270 nm or 1200 nm), and recorded by the multi-channel scaler (Nano-Harp 250, Pico-Quant, Berlin, Germany) for 60 s. We measured the decay curves at 1270 nm (singlet oxygen emission) and 1200 nm (background), and subtracted the background from the 1270 nm curves. The emission signal intensity was calculated by accumulating the intensity of the decay curve from 200 ns to 100 μs. The ^1^O_2_ quantum yields (φ_Δ_) of each dye in octanol were calculated by comparing the intensity of the signal of each dye to that of methylene blue in octanol. The intensity of methylene blue in octanol was, in turn, estimated from that in ethanol (0.520)^33^ by comparing the ^1^O_2_ emission intensities sensitized by methylene blue in octanol and in ethanol, which were compensated for light absorption, refractive index, and emission lifetimes. As a result, the ^1^O_2_ generation quantum yield of methylene blue in octanol was determined as 0.484.

### Measurement of fluorescence quantum yield of dyes

The quantum yields of the dye solutions were measured using an absolute photoluminescence quantum yields measurement system, Quantaurus-QY C11347-11 (Hamamatsu Photonics K.K.). The dye solution (2 mL) was set in a specialized quartz cuvette and excited at a wavelength shorter than the short-edge of the fluorescence spectrum.

### Evaluation of the correlation between endosomal escape efficiency and photophysical parameters

The relative efficacy of excitation (Ef) of each photosensitizer, which is relative to that of DY630 (Ef for DY630 = 1), was calculated using the spectrum of the excitation light source (Supplementary Fig. 3) and absorbance spectra of the photosensitizers (Ef = 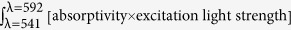. The values of [φ_Δ_ × Ef] and [φ × Ef] are linearly correlated to photogenerated ^1^O_2_ and fluorescence, respectively. The values of [(1 − φ −  φ_Δ_) × Ef] are considered to be proportional to the photogenerated heat. From these, we generated correlation plots of endosomal escape efficiency versus [φ_Δ_ × Ef], [φ × Ef], and [(1 − φ − φ_Δ_) × Ef].

### Cell culture, cell treatment with TatU1A-dye/RNA complexes, and photostimulation

Chinese hamster ovary (CHO) cells were cultured in Ham’s F-12 medium (Nacalai Tesque, Kyoto, Japan) supplemented with 10% fetal bovine serum (Nichirei Biosciences, Tokyo, Japan), 100 units/mL penicillin, and 100 μg/mL streptomycin (Gibco, Invitrogen, Carlsbad, CA). Treatment of the cells with TatU1A-dye/RNA complex was performed as follows: TatU1A-dye (2 μM) and the FAM-labeled shRNA (200 nM) were mixed in T buffer [20 mM HEPES-KOH (pH 7.4), 115 mM NaCl, 5.4 mM KCl, 1.8 mM CaCl_2_, 0.8 mM MgCl_2_, and 13.8 mM glucose] and incubated at 37 °C for 10 min. CHO cells were grown on a 96-well plate to 70–80% confluence and were treated for 2 h with the TatU1A-dye/RNA complex. After washing, the cells were visualized using a fluorescence microscope (Olympus, Tokyo, Japan). For endosomal escape of the TatU1A-dye/RNA complex, cells were irradiated for 2.6 sec with a 100 W mercury lamp (Olympus U-LH100HG) passed through the WIY mirror unit, a 40× objective lens (Olympus 40 ×/0.60 Ph2 LUCPlanFLN), and a 12% ND filter. This light source obtained with the WIY mirror unit provided peak light intensity at 580 nm. By this irradiation method, the light dose to the cells was 10 J/cm^2^. The photoinduced endosomal escape efficiencies of the FAM-labeled RNA with each TatU1A-dye were calculated by counting the number of cells in which FAM fluorescence was dispersed within the cytosol after photostimulation (N_F_) and the total cell number (N_T_) using FAM fluorescence and phase-contrast images from the same area. The endosomal escape efficiency was defined as N_F_ / N_T_ × 100 (%).

### Influences of ^1^O_2_ quenchers toward photo-dependent endosomal escape of the TatU1A-dye/RNA complexes

Cells were treated with TatU1A-Alexa633/RNA complexes as described above. One of the ^1^O_2_ quenchers listed below was added to the cells at the following time points; (i) at the same time as treatment with the complex, or (ii) after treatment with the complex. The ^1^O_2_ quenchers used here were L-histidine (final concentration 25 mM for incubation of the cells), NaN_3_ (25 mM), crocetin (50 μM), and α-tocopherol (50 μM).

### Imaging of photoinduced endosomal disruption by fluorescence microscopy

CHO cells were seeded on a 35-mm glass-bottom dish (MatTek, P35G-1.5-10-C) at a concentration of 2 × 10^4 ^cells per dish (the volume of medium was 150 μL per dish). On the next day, TatU1A-Alexa546/FAM-shRNA complexes prepared as described above in 100 μL T buffer were loaded onto the cells and incubated for 2 h in a CO_2_ incubator. The cells were washed with 150 μL T buffer twice and then observed in T buffer. Live cell imaging was performed using a DeltaVision microscope system (Applied Precision) placed in a temperature controlled room (37 °C)^34^. Light irradiation and subsequent time-lapse imaging were performed through the Olympus UApo/340 oil-immersion objective lens (40×, NA 0.65-1.35) at the NA value of =0.65. For the excitation of Alexa546, light at the wavelength of 529–556 nm (InsightSSI) was used at an irradiance of 1.47 W/cm^2^ for 6.8 s (approximately 10 J/cm^2^). Immediately after the irradiation, time-lapse images were obtained every 5 s. For measuring the fluorescence intensities at endosomal dots in the time-lapse images, ImageJ software (National Institutes of Health, Bethesda, MD) equipped with the SpotTracker 2D plug-in was used.

## Additional Information

**How to cite this article**: Ohtsuki, T. *et al.* The molecular mechanism of photochemical internalization of cell penetrating peptide-cargo-photosensitizer conjugates. *Sci. Rep.* **5**, 18577; doi: 10.1038/srep18577 (2015).

## Supplementary Material

Supplementary Information

## Figures and Tables

**Table 1:** Singlet oxygen quantum yield (φ_Δ_), fluorescence quantum yield (φ), and [1 − φ − φ_Δ_] of each dye in octanol, and photoinduced endosomal RNA escape efficiency with each TatU1A-dye.

Dye	Singlet oxygen quantum yield (φ_Δ_)	Fluorescence quantum yield (φ)	1 − φ − φ_Δ_*	Endosomal escape efficiency (%)
Alexa Fluor 594	0.028	0.922	0.050	60.6 ± 6.3
HiLyte Fluor 594	0.006	0.628	0.366	32.9 ± 7.3
DY 630	0.011	0.170	0.819	22.9 ± 4.3
Alexa Fluor 633	0.043	0.706	0.251	86.6 ± 3.9
DyLight 633	0.005	0.060	0.935	10.6 ± 1.6
Promo Fluor 633	0.012	0.305	0.683	24.8 ± 3.7
HiLyte Fluor 647	0.007	0.362	0.631	11.6 ± 1.8
Cy5	0.009	0.464	0.527	11.4 ± 2.8

^*^Photo-dependent heat generation efficiency.
