# Supplementary material for: The molecular mechanism of photochemical internalization of cell penetrating peptide-cargo-photosensitizer conjugates
Source: Sci Rep. 2015 Dec 21;5:18577. doi: 10.1038/srep18577 (PMC4685267; doi:10.1038/srep18577)
Supplement: Supplementary Information [file srep18577-s1.pdf]

Supplementary Information for

The molecular mechanism of photochemical internalization of cell penetrating peptide-cargo-photosensitizer conjugates

Takashi Ohtsuki, Shunya Miki, Shouhei Kobayashi, Tokuko Haraguchi, Eiji Nakata, Kazutaka Hirakawa, Kensuke Sumita, Kazunori Watanabe, Shigetoshi Okazaki

Methods

Synthesis of rose bengal-maleimide

General methods

Chemicals were purchased from Sigma-Aldrich (St. Louis, MO), TCI (Tokyo, Japan), and Wako (Osaka, Japan) and were used without further purification. Anhydrous solvents [dimethylformamide (DMF)] from Wako were used without further treatment and distillation. Thin layer chromatography (TLC) was performed on TLC-aluminum sheets (Silica gel 60 F254). Column chromatography was performed using Kanto Chemical Silica Gel 60 N (230–400 mesh) (Tokyo, Japan). ^{1}H NMR spectra were recorded on a JEOL JNM-ECP300 spectrometer (300 MHz) (Tokyo, Japan) with tetramethylsilane as the internal standard. Chemical shifts are reported in ppm. Mass spectra were recorded on a JEOL JMS-T100 instrument with electron spray ionization (ESI).

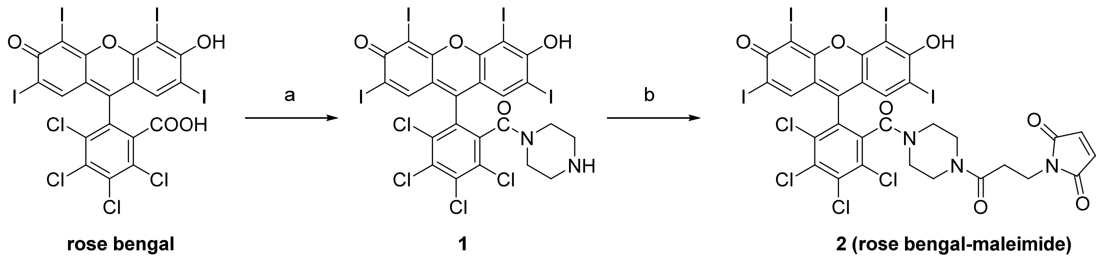

Scheme 1. Synthesis of rose bengal-maleimide. (a) piperazine, PyBOP, DMF, rt, 12 h, 73% yield; (b) N-Succinimidyl 3-maleimidopropionate, DIPEA, DMF, 37 °C, 6 h, 75% yield.

Synthesis of compound 1

Piperazine (12 mg, 140 μmol) and PyBOP (25 mg, 48 μmol) were added to rose bengal (43 mg, 44 μmol) in DMF (2 mL) at room temperature. The reaction mixture was stirred for 12 h, then the solvent was evaporated to dryness in vacuo. The crude products were dissolved in ethylacetate and the organic phases were washed with 0.1 M NaOH and brine and dried with sodium sulfate, filtered, and concentrated in vacuo. The crude was purified by HPLC on an ULTRON VX-ODS (0.1% TFA in H2O/0.1% TFA in CH3CN : 55:45 to 5:95 (v/v) for 40 min) to afford a 40% (18 mg, 17 μmol) yield of product 1 as a purple solid.

^{1}H-NMR (300 MHz, CD_3OD) δ 7.64 (s, 1H), 7.50 (s, 1H), 3.18-3.12 (m, 2H), 1.87-1.83 (m, 2H) ESI-TOF-MS *m/z* calc. for $C_{24}H_{11}Cl_4I_4N_2O_4$ 1040.56, found 1040.43 $[M-H]^-$

Synthesis of compound 2 (rose bengal-maleimide).

N-Succinimidyl 3-maleimidopropionate (10 mg, 38 μmol) was added to Compound 1 (7 mg, 6.7 μmol) in DMF (2 mL) and DIEA (20 mg, 144 μmol) at 37 °C. The reaction mixture was stirred for 6 h, then the solvent was evaporated to dryness in vacuo. The crude was purified by column chromatography on silica gel ($CHCl_3$/MeOH =10:1 (v/v)) to afford a 75% (6 mg, 5.0 μmol) of product 2 (rose bengal-maleimide) as a purple solid.

^{1}H-NMR (300 MHz, CD_3OD) δ 7.73 (s, 1H), 7.32 (s, 1H), 6.99-6.98 (m, 2H), 3.57-3.51 (m, 2H), 2.59-2.50 (m, 10H)

ESI-TOF-MS *m/z* calc. for $C_{31}H_{16}Cl_4I_4N_3O_7$ 1191.60, found 1191.42 $[M-H]^-$

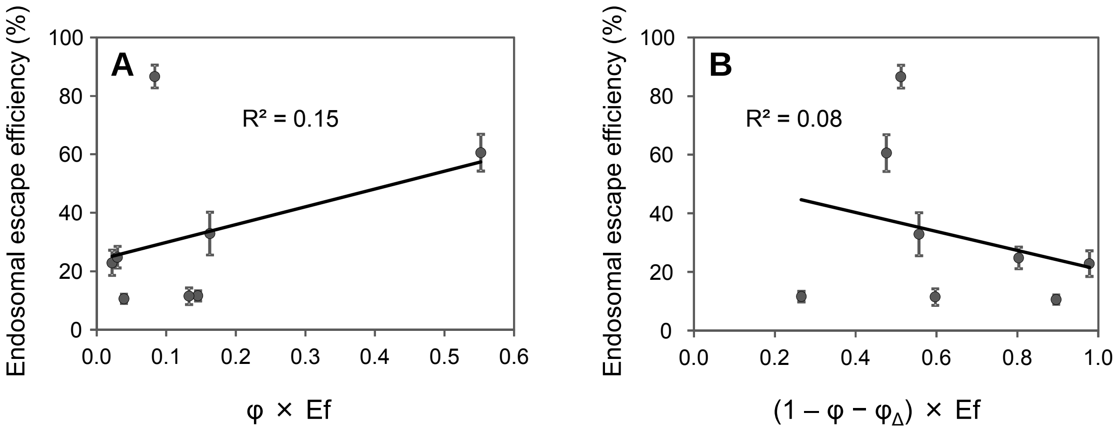

Supplementary Fig. S1. Correlations between photoinduced endosomal escape efficiencies of TatU1A-dye/RNA complexes and [φ × Ef] (**A**) or [(1 – φ – $φ_Δ$) × Ef] (**B**) of each dye in H_2O including 50 mM NaOAc (pH 5.5). Data represent means ± SD, n = 5.

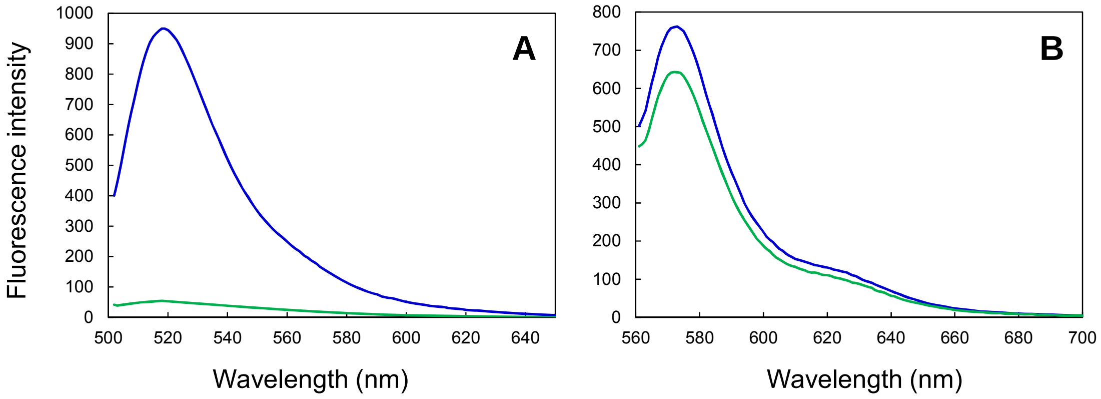

Supplementary Fig. S2. Fluorescence spectra of FAM-labeled shRNA (**A**) and Alexa Fluor 546 (**B**) measured in 1 mM HEPES-KOH (pH7.2) (blue) and 1 mM NaOAc (pH 5.5) (green). The concentrations of FAM-labeled shRNA and Alexa Fluor 546 were 400 nM and 3 nM, respectively. These spectra were measured using a JASCO FP-6600 spectrofluorometer (Easton, MD). The excitation wavelengths were 485 nm for FAM and 545 nm for Alexa Fluor 546.

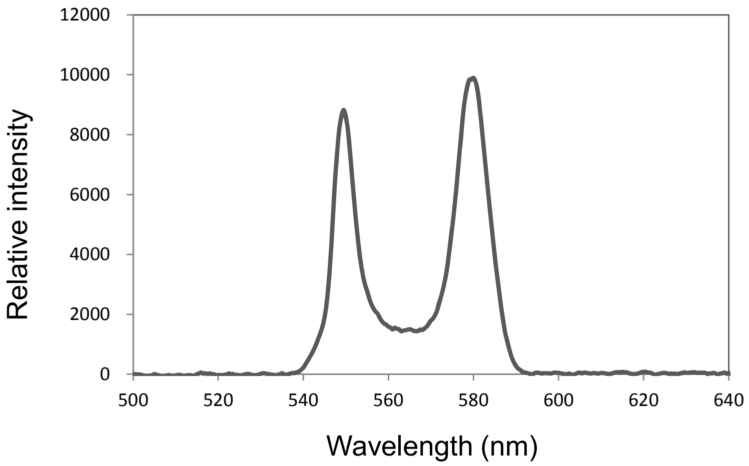

Supplementary Fig. S3. The spectrum of light emitted by the mercury lamp (Olympus U-LH100HG) through the WIY mirror unit. This spectrum was measured using an USB4000 spectrometer (Ocean Optics).
